# Medical Etiquette and Professional Rights

**Published:** 1856-08

**Authors:** 


### MEDICAL ETIQUETTE AND PROFESSIONAL RIGHTS.

According to Judge Minot, of Pennsylvania, (whose views have been, we believe, already published in this Journal,) and according to common sense, "It is a rule of law, that a medical practitioner never insures the result, but simply engages that he possesses a reasonable degree of skill, such as is ordinarily possessed by a profession generally, and to exercise that skill with reasonable care and diligence; and again, to exercise his best judgment, *but is not responsible for a mistake of judgment;* beyond this, the physician is not responsible: these are received, in general, as sound views, and such as will govern every enlightened court. There could scarcely be a greater absurdity than to require physicians and surgeons to insure the result, when they can in no case control all parts of the treatment. Few serious cases are carried through a single day, and many not a single hour, without a violation of instructions upon the part of nurses and attendants." But it is not in a legal point of view that we propose to consider this subject. There seems to be in this locality, and perhaps in some other sections, an entire want of appreciation upon the part of the public of what we conceive to be the *rights* of physicians.

We have no hesitation in asserting that when a medical man is entrusted with the management of a case of disease, it is with the implied understanding that he shall control it to the termination, with the implied engagement upon his part, that he possesses an ordinary amount of skill—that he will attend it with reasonable care and diligence, and bring to bear upon it his best judgment.

Unless, therefore, the practitioner in charge can be convicted of gross negligence or ignorance, the individual who employs him, has no good reason for dissatisfaction, and any manifestation of discourtesy or want of confidence that would induce him to retire, is unbecoming, highly censurable, and violative of the self-imposed obligations under which he entered when soliciting the services of the physician. Under the circumstances indicated, an ordinary amount of skill and attention being furnished, the individual has an all-sufficient remedy for whatever anxiety he may feel in reference to the result of the case, in the acknowledged right to have consultation: to this, no true medical man will object; so far from it, the division of responsibility and the desire to benefit his patient, by any and all means, will induce his ready and hearty assent upon the slightest intimation of such a desire.

And while, of course, no medical man would be willing to retain his position as physician, where he was not satisfied that his services were acceptable, and would immediately surrender a patient upon the slightest manifestation of dissatisfaction or want of confidence, we still contend most strenuously for what we believe to be the *rights* of the profession, being satisfied, if they were better understood upon the part of the people, and more decidedly maintained upon the part of medical men, that it would be greatly to the interest of both: our opportunities for treating disease successfully would be greatly increased, and we should much less frequently have unpleasant relations among the members of our profession.

Who but some *bastard* doctor would be willing to undertake the treatment of disease, with the understanding that it was a mere *trial* of his skill, and in the event of a failure upon his part to remove the malady, in a time fixed by those who solicited his services (of course, utterly ignorant of the whole matter, and incompetent to decide upon the period required for its management) he was expected to retire, or with the full knowledge of the fact, that he was completely at the mercy of the whims and caprices of those who employed him, and liable to be subjected to discharge at any moment, without, in many cases, having had the time indispensably required for the removal of the malady, and that, too, without any allowance for the innumerable circumstances that may arise to render the best efforts of the physician abortive, and with the entire absence of the recollection (so often witnessed upon the part of the public) *that people must die?*

No honorable member of our profession, we are sure: this, then, we believe to be a fair statement of the case, and therefore we contend that unless the physician manifest gross ignorance, negligence, or moral delinquency, the individual who employs him has no *right* to discharge him, (the term right, as will be manifest, being used with reference to what is just and proper,) directly or indirectly; and according to our view, the control of this whole matter rests with the profession, and no man ought to allow himself to be made the instrument of violating one of the most sacred and important rights of our calling, by consenting to assume the management of a patient who has been recently in the charge of another, unless the case has been *voluntarily* surrendered by his professional brother, or under circumstances where the services of the practitioner in attendance, cannot be obtained. As a matter of policy alone, it seems to us that the rigid observance of this rule is required; for upon the same principle, the second and the third are discharged, as it may suit the notions of the public, and thus we may have a case *bobbing* the rounds of the profession (no one having had a fair opportunity of treating the case) engendering jealousy and ill feeling, and in every way bringing discredit upon our calling. That this most objectionable state of things exists to a large extent, we presume no one will deny, as it is a notorious fact, that in this locality at least, you frequently have a case of disease transferred without ceremony and at discretion, and in some cases without even notifying the physician last called in, that it had been subjected to previous treatment.

This condition of affairs we have reason to know is generally lamented by the profession, and is only submitted to, upon the false supposition, that there is no remedy, and the only reflection which we design to cast upon those who are suffering is, *that they do not, by a united and organized movement, asssert and maintain their rights*, for we are sure that there is no true medical man to be found any where, who would be deterred by any consideration connected with fear of personal loss or injury, from taking a stand for any principle important to the honor or interest of medicine.

